# From Immunosuppression to Immunomodulation - Turning Cold Tumours into Hot

**DOI:** 10.7150/jca.71992

**Published:** 2022-07-04

**Authors:** Mariangela Garofalo, Katarzyna Wanda Pancer, Magdalena Wieczorek, Monika Staniszewska, Stefano Salmaso, Paolo Caliceti, Lukasz Kuryk

**Affiliations:** 1 Department of Pharmaceutical and Pharmacological Sciences, University of Padova, Via F. Marzolo 5, 35131 Padova, Italy; 2 Department of Virology, National Institute of Public Health NIH —National Research Institute, Chocimska 24, 00-791 Warsaw, Poland; 3 Centre for Advanced Materials and Technologies, Warsaw University of Technology, Poleczki 19, Warsaw 02-822, Poland

**Keywords:** oncolytic virus, TILs, immunotherapy, mesothelioma, melanoma, drug delivery

## Abstract

Cancer cells employ various mechanisms to evade and suppress anti-cancer immune responses generating a “cold” immunosuppressive tumour microenvironment. Oncolytic viruses are a promising tool to convert tumour immunosuppression to immunomodulation and improve the efficacy of cancer treatment. Emerging preclinical and clinical findings confirm that oncolytic viruses act in a multimodal scheme, triggering lyses, immunogenic cell death and finally inducing anti-cancer immune responses. In this paper, we tested the local administration of a novel oncolytic adenovirus AdV-D24-ICOSL-CD40L expressing co-stimulatory molecules ICOSL and CD40L to induce the production of tumour infiltrating lymphocytes to the site of injection. Subsequently, in immunocompetent mouse models, we studied possible correlation between tumour infiltrates and anti-cancer efficacy. Described results showed that the delivery of oncolytic viruses encoding immunomodulatory transgenes in combination with anti-PD1 resulted in synergistic inhibition of both melanoma and mesothelioma tumours. Importantly anti-cancer effect positively correlated with cytotoxic CD8+ tumour-infiltrating lymphocytes exerting a central role in the tumour volume control thus generating beneficial outcomes that will undoubtedly provide new insights into possible future treatment strategies to combat cancer. Altogether our findings highlight the importance of oncolytic vectors able to modulate anti-cancer immune responses that can correlate with efficacy in solid malignancies.

## Introduction

The tumour-infiltrating lymphocytes (TILs) is represented by a subgroup of the heterogeneous group of immune cells including mono- and poly-morphonuclear cells of the innate immunity such as NKs, macrophages, DCs, mastocytes, basophils, neutrophils, eosinophils and adaptive T and B cells 1. Over the years it has become clear how important it is to analyse the nature of the TILs in the tumour microenvironment (TME). In fact, the ratio between anti-tumour immune cells and the immune-suppressive cells infiltrated in the TME is correlated with the prognosis of cancer 2,3. More precisely, when an immune activator agent reaches the tumour tissue an immune response is triggered, aiming to enhance tumour-specific TILs' infiltration. Consequently, the TME becomes progressively more and more inflamed, overcoming the immune-suppressive conditions that would allow the tumour's immune surveillance escape.

There is increasing evidence supporting a role for immunotherapy in solid tumours such as melanoma and malignant pleural mesothelioma (MPM). Sobhani et al. reported that tumours with mild levels of TILs from MPM patients with epithelioid histological type markedly correlated with improved survival compared to tumours with absent or low TILs expression 4. Further concerning melanoma, a longer survival was observed in melanoma patients presenting high density of CD8+ cytotoxic T cell infiltration, while in contrast, the infiltration of immunosuppressive tumour-associated macrophages (TAMs) and Treg cells within the tumour has proven to have a negative impact on tumour progression 5,6. These results stressed the relevance of immunotherapy in cancer treatment. However, it's important to distinguish between T-cell inflamed (“hot”) and non-T-cell inflamed (“cold”) TME, relying on the presence or absence of TILs, which are considered as fundamental biomarkers for solid tumours 7,8. Beside the T cell infiltration level, also the amount and variety of pro-inflammatory cytokine is a relevant factor, and as a matter of fact hot tumours are classified by molecular signatures of immune activation, differently from the cold tumours 9. As a consequence, intensive efforts have been invested in the development of novel strategies aiming to allow the “cold- to-hot” conversion of the TMEs, to improve the outcome of the disease for patients treated with immunotherapeutic drugs 10. A cold tumour, in fact, represents a non-immunoreactive environment, and it has been associated to a scarce or absent production of effector tumour-specific T-cells, and consequently, patients bearing these tumours don't respond to anti- immune checkpoint inhibitors, since the drug target is missing, or is not involved in the process of immune cells inactivation 8. Collectively, tumorigenesis process is not related only to tumour cell characteristics but is deeply influenced by the TME. It is not surprising, therefore, how currently several different therapeutic strategies aim at inflaming the cold TMEs by increasing the lymphocytic infiltration in the tumour core and thus, leading to a better clinical prognosis.

A promising approach that is currently tested in various clinical studies consist of a novel class of innate immunity activators called oncolytic viruses (OVs), such vectors able to cause the infection and the lysis of cancer cells, spearing the healthy ones 11-13. It has been shown that virotherapy can modulate anti-cancer immune responses that enhance the efficacy of check-point inhibitors (CPI). Therefore, the combination therapies of oncolytic vectors with CPIs are an encouraging regime for cancer treatment. Furthermore, the efficacy of combining OVs plus CPIs has been shown in pre-clinical studies, and there are currently many ongoing clinical trials assessing combination therapies with inspiring findings 14. Nevertheless, despite extensive research, oncolytic viruses have shown limited efficacy against solid tumours as monotherapy 15. Therefore, the advancement of novel and more powerful oncolytic vectors is needed. We have previously engineered an innovative AdV-D24-ICOSL-CD40L able to encode for two co-stimulatory molecules ICOSL and CD40L 16. Upon cell lysis, both ICOSL and CD40L are released and act as soluble molecules, modulating the host anti-cancer activity by binding to their targets respectively activating the T cells and the APCs 16. The process of cell lysis induced by the novel OVs may lead to the elimination of the primary tumour and distant metastases, thus the infiltration of TILs in the tumour core, is enhanced, leading to the inflammation of the TME. Therefore, OVs can be able to break the cancer immune tolerance, encouraging also, the development of an immune memory against tumour antigens, which prevents further tumour recurrence.

To date, many oncolytic viruses are under development, at different phases of preclinical and clinical trials 11,17-20. A fundamental milestone in the development of oncolytic virotherapy for the management of advanced melanoma is Talimogene laherparepvec (T-VEC), under the trade name Imlygic 21. T-VEC is the first oncolytic virus approved for melanoma metastasis, with a reported overall response rate (ORR) of 25% and complete response rate (CRR) of 10% 22. Promising oncolytic adenovirus is called ONCOS-102, which is a chimeric 5/3 capsid expressing GM-CSF 23,24. The treatment with the virus in Phase I clinical study was able to induce anti-tumour immunity and signals of clinical efficacy 25. Another novel oncolytic virus called Coxsackievirus A21 (CAVATAK) exhibited synergy when administered with immune checkpoint inhibitors. A clinical trial evaluating CAVATAK in combination with ipilimumab resulted in 50% objective responses in melanoma patients 26. Promising results have been also reported in treatment with DNX-2401, where dramatic responses with long-term survival in recurrent high-grade gliomas were reported 27. Finally, VALO-D102 is a novel oncolytic adenovirus, expressing OX40L and CD40L, used in PeptiCRAd cancer vaccine platform. Intratumoral administration of PeptiCRAd profoundly elevated tumour-specific T cell responses, inhibited tumour growth, and activated systemic anti-cancer immunity in tested mouse models of melanoma. The combination of PeptiCRAd with anti-PD1, significantly improved anti-cancer effect 28.

Herein, the objective of this research was to assess the efficacy of administration of AdV-D24-ICOSL-CD40L in combination with anti-PD1 and their ability to generate infiltration of TILs. Moreover, we investigated possible correlation between the altitude of infiltration and anti-cancer effect- tumour volume and tumour weight- in tested immunocompetent melanoma and mesothelioma mouse models. Our results demonstrated that the local delivery of the oncolytic virus encoding immunomodulatory transgenes resulted *in vivo* synergistic anti-cancer effect in mice with established AB12 and B16V tumours. This study showcases the possibility of using a novel OV-formulation to induce the production of tumour-reactive TILs.

## Materials and Methods

### Cell lines, Virus, Anti-PD1 antibody

Juvenile Fibroblasts, isolated from human male foreskin 29-31 and B16V mouse melanoma cells were kindly provided by Prof. Rinner from the Medical University of Graz (Austria), while mouse mesothelioma cell line AB12 was obtained from Cell Bank Australia. Juvenile fribroblasts were cultured in in Dulbecco's Modified Eagle Medium (DMEM), 1% L-glutamine (Gibco Laboratories), 1% of penicillin/streptomycin (Gibco Laboratories), and 10% fetal bovine serum (FBS, Gibco Laboratories). Murine cell lines were cultured in RPMI 1640 medium (Gibco Laboratories, USA) supplemented with 1% of penicillin/streptomycin (Gibco Laboratories), 1% L-glutamine (Gibco Laboratories) and 10% FBS (Gibco Laboratories). The adenovirus vector AdV-D24-ICOSL-CD40L used in this work has a chimeric serotype 5/3 adenovirus and was generated and amplified using standard adenovirus preparation techniques as previously described 16. For this experiment a surrogate of the vector encoding mouse CD40L and ICOSL was used. The transgenes expression was induced by a CMV promoter (a CMV-ICOSL-IRES-CD40L expression cassette inserted in place of the E3 region). Purified anti-mouse CD279 (PD1) antibody has been resuspended according to manufacturers' instructions (BioLegend).

### CAR and DSG2 Expression in Cancer Cell Lines

AB12 and B16V cells were stained firstly with mouse monoclonal anti-CAR antibody (Santa Cruz Biotech, Dallas, TX, USA) and then with 1:2000 Alexa-Fluor 488 secondary antibody (Abcam, Cambridge, UK) or mouse monoclonal anti-DSG2 antibody (Abcam, Cambridge, UK) and then with 1:2000 Alexa-Fluor 488 secondary (Beckman-Coulter Cytomics FC500) (at least 10^4^ cells/events were analyzed by flow cytometry).

### Cell Viability: MTS Cytotoxicity Assay

Juvenile fibroblasts, B16V and AB12, were seeded at a density of 1 × 10^4^ cells/well in a 96-well plate and maintained under standard growth condition (DMEM or RPMI 1640, completed with 5% FBS, 1% L-glutamine and 1% of penicillin/streptomycin (all from Gibco Laboratories). After overnight incubation, cells were treated as follows: (i) PBS, (ii) AdV-D24-ICOSL-CD40L (0.1, 1, 10, 100 VP/cell). All treatments have been diluted in growth media with 2% FBS and cells were then incubated in 5% FBS containing media. Cell viability was determined 96 hrs after treatment, by using CellTiter 96 AQueous One Solution Cell Proliferation Assay (MTS) according to the manufacturer's instructions (Promega, Madison, WI, USA). The absorbance was measured with a 96-well plate spectrophotometer (Victor NivoTM, PerkinElmer, Milano, Italy) at 490 nm. The experiments were independently carried three times and each experiment contained triple replicates.

### In vivo efficacy studies

All animal procedures were performed and approved by the Austrian Federal Ministry of Science and Research (BMWF) (GZ 66.010/0058-V/3b/2019) and Italian Ministry of Health (117/2020-PR). For the efficacy experiments, melanoma murine xenografts were established by subcutaneously (s.c.) injecting respectively 1 × 10^6^ B16V cells into both flanks of 10-week-old C57BL/6 male mice (6 tumours/group) while mesothelioma murine xenografts were established by subcutaneously (s.c.) injecting respectively 1.5 × 10^7^ AB12 cells into both flanks of 10-week-old Balb/c male mice (6-10 tumours/group). Tumours (two tumours per mouse, ~5 × 5 mm in diameter) were randomized prior the treatment initiation as follows: mock (100 μL of PBS) administered intratumorally (i.t.), AdV-D24-ICOSL-CD40L administered i.t. at a concentration of 1.75 × 10^10^ VP/tumour (3.5 × 10^10^ VP/mouse), murine anti-PD1 (purified anti-mouse CD279 (PD1) antibody BioLegend) administered intravenously (i.v.) (200 μg/mouse), AdV-D24-ICOSL-CD40L administered i.t. at a concentration of 1.75 × 10^10^ VP/tumour (3.5 × 10^10^ VP/mouse) followed by i.v. treatment with anti-PD1 (200 μg/mouse) (Table 1). Tumour size was recorded using calliper on two dimensions every three days. The longest and shortest diameter of tumour at each timepoint were recorded and the tumour volume was calculated using a formula of 0.52 × length x (width)^2^. All animals were observed for clinical signs, morbidity, or mortality daily during the acclimatization and administration period and additionally 30 min after each treatment (Table S1). Characteristics of clinical signs in animal health scoring.

### Immune cell infiltrates

The percentage number of mouse immune cell populations were monitored by flow cytometry: mouse CD45+ (cat. number: 550994, BD) lymphocytes: whole T cells (mCD3+ (cat. number: 561798, BD), CD4+ T cells (mCD3+ hCD4+ (cat. number: 552775, BD), CD8+ T cells (mCD3+ mCD8+ (cat. number: 560182, BD). Tumours were harvested and subsequently dissociated with cell strainer. Immune cells were isolated by following the protocol described earlier 32. After dissociation, cells were washed and stained with antibodies 30 min at 4ºC in the dark and then suspended in PBS. Samples were acquired using BD FACSAriaTM III instrument. The populations were gated with forward and side scattering (FSC-A/SSC-A dot plot) in leukocytic regions. Flow cytometry analysis was performed on FlowJo v10 software.

### Histopathological studies

Tissues from murine tumour, spleen and liver underwent routine paraffin processing followed by sectioning at 4 μm and staining with Hematoxylin and Eosin (HE). The histopathological evaluation was performed as a blind using 40×magnification using the microscope Zeiss Axio Imager A2, Carl Zeiss Microscopy GmbH, Jena, Germany.

### Statistical Analysis

GraphPad Prism software (Version 9) was used to analyse *in vivo* variables. Statistical analysis was comprised of a repeated measures one way ANOVA test. The FTV calculation method was used to assess therapeutic synergism 17,18,33. Briefly, the observed FTV equated the mean tumour volume for each experimental group divided by the mean tumour volume of the PBS control group. The expected FTV for a combination (AdV-D24-ICOSL-CD40L plus anti-PD1) equals the product of the observed FTV for the individual groups (expected combinatory therapy = FTV_AdV-D24-ICOSL-CD40L_* FTV_anti-PD1_). The ratio of the expected FTV divided by the observed FTV indicated the nature of the interaction: >1 indicated synergism, 1 indicated additive, and < 1 indicated less than additive (antagonism). Pearson correlation coefficient was used to identify potential relationships between tumour volume and percentage of CD4+ and CD8+ T cells in the tumour infiltrating lymphocytes in tumour tissue.

## Results and Discussion

Oncolytic virotherapy is a promising and game-changing cancer treatment, getting increasing interest among researchers worldwide. In the field of cancer immunotherapy, the immunological profile of TME is a key determinant of disease prognosis and therapeutic outcome. Based on reported results it has been shown that OVs are able to modulate and alter TME landscape, resulting in enhanced anti-cancer activity alone or in a combination with other agents. Therefore, OVs are potent immune activators able to promotes profound, long-lasting anti-cancer immune responses and clinical efficacy 33-36.

In this paper, we investigated the ability of novel oncolytic adenovirus AdV-D24-ICOSL-CD40L expressing co-stimulatory molecules ICOSL and CD40L 16 to induce infiltration of TILs to the site of injection. Subsequently, in tested animal models, we evaluated possible correlation between tumour infiltrates and anti-cancer efficacy. Therefore, a central hypothesis of our study was that the intratumoral treatment with the novel oncolytic adenovirus AdV-D24-ICOSL-CD40L combined with CPIs can reshape TME by enhancing infiltration of TILs and inducing immune response against tumour, which will then correlate with clinical efficacy.

**Figure 1:**
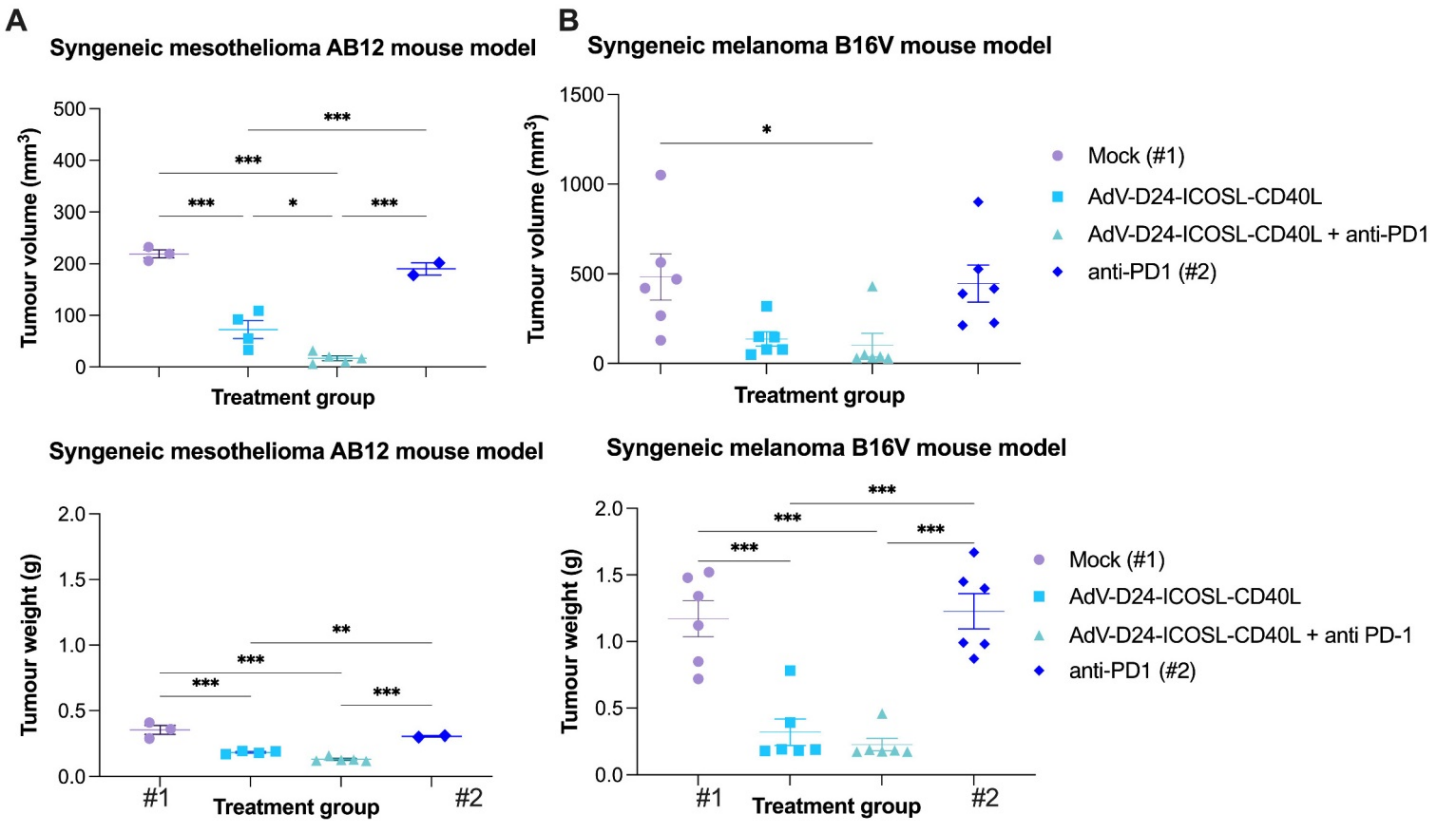
Anti-cancer properties of tested agents in immunocompetent mouse models. A BALB/c AB12 xenograft mesothelioma mouse model. Mice were engrafted with 1x10^6^ cells/flank. The virus was administered intratumorally on days 1-6 i.t, anti-PD1 was given i.p. on days 1-6. The tumour volumes and weights, clinical health scores were monitored 2-3 times per week. At the end of the study mice were euthanized and tumour collected for immunological analyses. The average tumour volumes and weights are presented as mm^3^ ± SEM. B C57BL/6 B16V xenograft melanoma mouse model. Mice were engrafted with 5x10^6^ cells/flank cells/flank. The virus was administered intratumorally on days 1-6 i.t, anti-PD1 was given i.p. on days 1-6. The tumour volumes and weights, clinical health scores were monitored 2-3 times per week. At the end of the study mice were euthanized and tumour collected for immunological analyses. The average tumour volumes and weights are presented as mm^3^ ± SEM. #1 mice in mock group, #2 mice in anti-PD1 treated group (C57BL/6), have been euthanized due to ethical reason before end of the study (tumour volume exceeded 1000 mm^3^). Therefore, the latest available tumour volume/weight measurements have been considered. Results are expressed as mean ± SEM. ANOVA was used. * P ≤ 0.05; ** P ≤ 0.01; *** P ≤ 0.001.

Firstly, the oncolytic properties of AdV-D24-ICOSL-CD40L have been evaluated through the MTS cell viability assay (Figure S1). We assessed the overall tumour cell selectivity of the novel oncolytic adenovirus on non-tumour cells juvenile fibroblasts. The results show that none of the tested conditions displayed an impact on the growth of non-tumour cells (cell availability > 90%), confirming the tumour-cell selectivity of the virus. In contrast, cell viability experiments carried out on B16V and AB12 demonstrated that tested virus was able to infect and induce cell death in those cell lines, especially at a concentration of 100 VP/cell (cell viability was 40.4% and 44.7% for B16V and AB12, respectively) (Figure S1). This is not surprising as it is known that genetically modified adenovirus, delta-24, which has a 24-base pair deletion in the Rb-binding region of the E1A gene, shows selective replication and oncolysis in various malignant cells 37,38. Expression level of adenovirus cell entry receptors on the surface of cancer cells show that the B16V and AB12 cell line expressed DSG2 receptors (approximately 21% and 98% of cells positive for the marker respectively). The expression of CAR receptors low in B16V (negative for CAR expression) compared to AB12 (approx. 90% (Figure S2), thus, suggesting that both cancer cell lines can be targeted with oncolytic adenoviruses exhibiting affinity to DSG2 receptors for therapeutic treatment 22. Subsequently, to evaluate the ability of AdV-D24-ICOSL-CD40L alone or in combination with anti-PD1 to induce infiltration of TILs we established immunocompetent melanoma and mesothelioma mouse models in syngeneic tumour models: C57BL/6 and BALB/c mice. In our previous preclinical study, we tested anti-cancer efficacy of oncolytic adenovirus expressing GM-CSF in humanized mice engrafted with A2058 melanoma cells. Our previous results reveal that the combination of anti-PD1 with the virus significantly reduced tumour volume and exhibited synergistic anti-cancer effect 39. Herein, *in vivo* research was carried out to investigate the possible anti-tumour effects triggered by the combination therapy according to the scheme listed down in Table 1. We showed that the therapy with the virus in combination with anti-PD1 was the most effective regimen in both tested animal models. At the end point (day 33), mesothelioma tumour volumes of mice treated with the combinatory therapy were markedly smaller compared to the other ones treated virus alone or in mock group (9,9 mm^3^, 72 mm^3^, 219 mm^3^, p≤ 0.001, respectively for the combination therapy, virus, and mock group) (Figure 1A). The therapy with anti-PD1 did not significantly reduce the tumour volume (vs mock). Similar observations have been reported in melanoma B16V model, where the combination treatment of AdV-D24-ICOSL-CD40L + anti-PD1was superior over other treatments (102 mm^3^, 137 mm^3^, 484 mm^3^, p≤ 0.001, respectively for the combination therapy, virus, and mock group) (Figure 1B). In line with previous findings also in this model, the therapy with anti-PD1 did not exhibit anti-cancer efficacy.

No significant changes in body weight have been reported thorough the study (Figure S3). Interestingly, mice bearing B16V melanoma tumours and treated with combination scheme (Table 1), exhibited 100% survival thus suggesting its safe profile (data not shown). In contrast mice in mock and anti-PD1 treated group in C57BL/6 mice have been euthanized due to ethical reason before end of the study (tumour volume exceeded 1000 mm^3^). In fact, the B16 is one of the most aggressive melanoma cell lines for C57BL/6 40. This cell line is highly aggressive and can metastasize from a primary subcutaneous site to the lungs 41. Therefore, this model allowed us to test the anti-cancer efficacy of the combinatory approach in highly aggressive skin cancer.

It has been previously reported that the use of OVs in combination with chemotherapy has resulted in synergistic anti-cancer interactions in mesothelioma BALB/c nude mouse model 17. Further research also demonstrated that OVs given together with doxorubicin or cisplatin plus carboplatin resulted in synergistic anti-tumour activity against soft-tissue sarcoma in Syrian hamster 42. Moreover, Zhang et al. described that oncolytic viruses synergistically enhanced anti-PD-L1 and anti-CTLA-4 immunotherapy by reshaping the TME 43. Therefore, to check this possibility, we performed fractional tumour volume analyses which revealed that the combination therapy resulted in synergistic anti-cancer effect in both cancer models (3.7 and 1.2 FTV ratio for mesothelioma and melanoma models respectively) (Table 2), thus suggesting the potential of the proposed approach for improved anti-cancer efficacy and induction of antineoplastic immunity.

**Figure 2:**
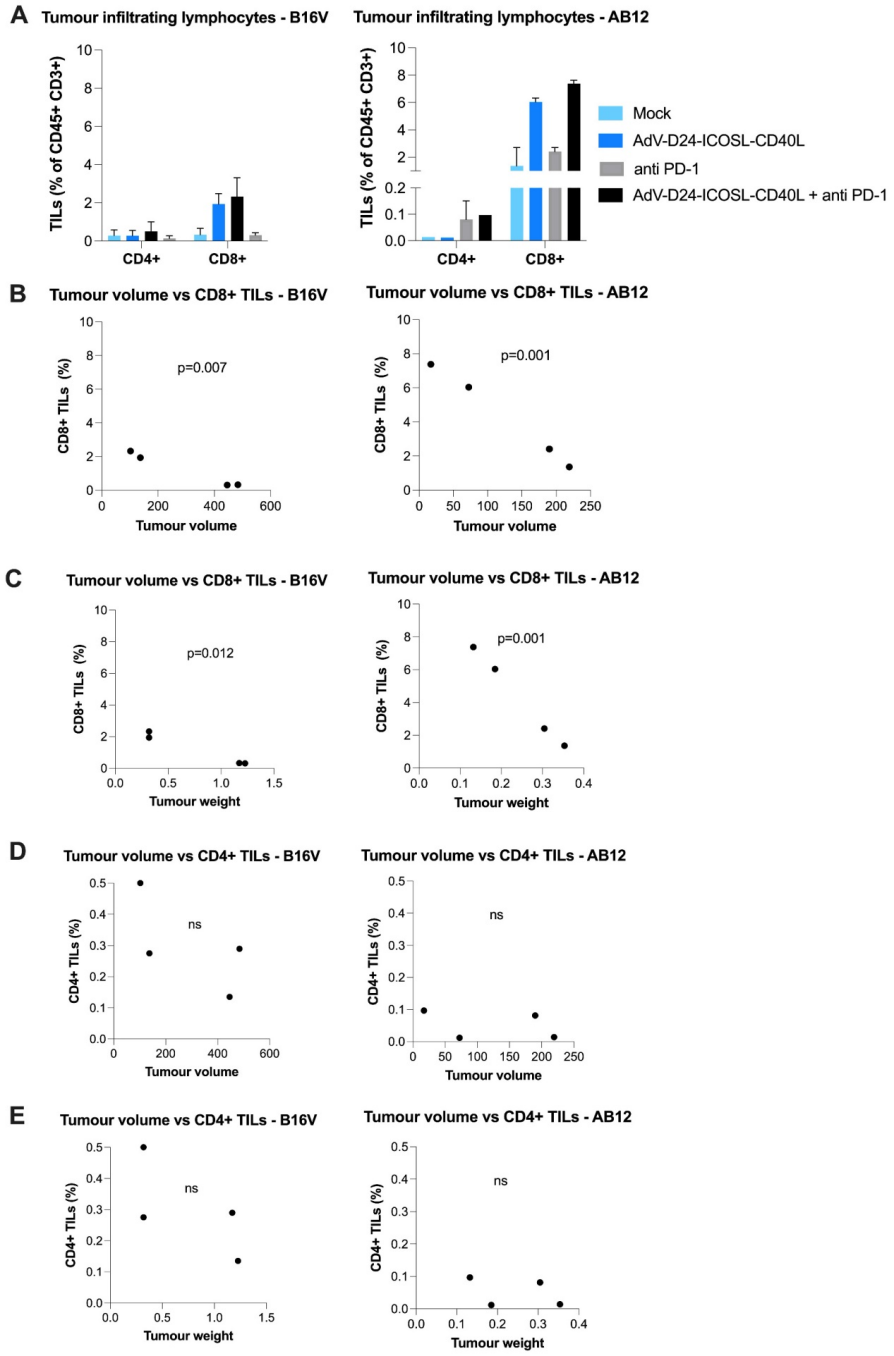
Anti-cancer and immunomodulatory properties of tested agents in immunocompetent mouse models. At the end of the study mice were euthanized and tumour collected for immunological analyses (the latest available tumour samples have been collected for TILs isolation). **A** Tumour infiltrating lymphocytes CD4+ and CD8+ expression has been assessed in collected tumours. The populations were gated with forward and side scattering (FSC-A/SSC-A dot plot) in leukocytic regions (analysed by flow cytometry, 6-10 tumours/experimental group). **B-E** Pearson correlation coefficient was used to identify potential relationships between tumour volume/tumour weight and percentage of CD4+ and CD8+ T cells in the tumour infiltrating lymphocytes in tumour tissue. Individual data and mean +/- SEM are presented for each group. Results are expressed as mean ± SEM. ANOVA was used. * P ≤ 0.05; ** P ≤ 0.01; *** P ≤ 0.001.

Considering that advanced carcinomas are often associated with impaired immune recognition and a highly immunosuppressive TME, the presence of TILs prior a treatment relates to a good clinical prognosis, while the opposite is linked to TME immunosuppression 44. Despite the importance of both CD4+ and CD8+ T cells subsets, the different effector function of these adaptive immunity cells makes fundamental the characterization of the TILs' composition within the tumour mass, which might represent an important prognostic factor. Focusing on the results obtained on B16V and AB12 model, concerning the infiltration of CD4+ helper and cytotoxic CD8+ T cells it's possible to see how the tested treatment induced an infiltration. Phenotyping analyses of TILs isolated from collected tutors in both animal models showed that the virus alone or combined with anti-PD1 was able to increase the level of immune cell infiltrates, especially CD8+ T cells (AB12: mock: 1.36%, virus: 6%, anti-PD1: 2.4%, combination therapy: 7.38%; B16V: mock: 0.33%, virus: 1.94%, anti-PD1: 0.31%, combination therapy: 2.33%) (Figure 2, Figure S4). Collectively, looking at these results it is possible to speculate that this novel chimeric adenovirus is effectively able to enhance the infiltration of CD8+ cytotoxic T lymphocytes, and therefore exerting a central role in the tumour volume control. Our results show that CD8+ TILs (but not CD4+) count significantly correlated with the tumour volume (p=0.001, p=0.007 respectively for AB12 and B16V models) (Figure 2B) and the tumour weight (p=0.001, p=0.012 respectively for AB12 and B16V models) (Figure 2C). These findings suggest that AdV-D24-ICOSL-CD40L therapy sensitizes tumours to other immunotherapies (e.g. CPIs).

In point of fact, in Phase I clinical study (NCT01598129) the therapy with oncolytic virus ONCOS-102 (AdV5/3-D24-GM-CSF), in patients resistant to available treatments resulted in 40% of disease control rate in evaluable patients (4/10) at 3 months and median overall survival (mOS) was over 9 months. A sound infiltration of TILs to tumour lesions was reported post-treatment in 11 out of 12 patients. Similarly, correlation between cytotoxic CD8+ TILs and macrophages CD68+ in tumours and overall survival was reported, suggesting that the virus was able to reshape local immunological TME at tumours and recruit activated cytotoxic CD8+ T cells able to control disease progression 25,45. In line with this observation other study evaluated the prognostic role of CD8+ TILs in cancer patients treated with checkpoint inhibitors. Obtained results suggested that high intertumoral infiltration but not circulating cytotoxic T cells, can foresee treatment outcomes in a population with checkpoint inhibitor therapy across different cancer indications 46.

Furthermore, histological analysis showed that samples treated with the combination therapy exhibited no significant histopathological findings: normal liver lobule and normal spleen (Figure S5). Indeed, tumour samples from mice treated with AdV-D24-ICOSL-CD40L plus anti-PD1 show necrotic remnants of cells in a small necrotic area and apoptotic cell remnants in degenerative area, with profound presence of immune cell infiltrates, while tumours treated with PBS showed less frequent presence of immune cell infiltrates with lower number of apoptotic/necrosis cells.

Nevertheless, despite these findings, further research aiming at more detailed phenotyping analyses of TILs, including activation and exhaustion markers is required to better understand how anti-tumour immune responses induced locally, contributes to clinical systemic efficacy. Investigation of tumour antigen specific T cells from the peripheral blood can also provide supportive findings. Although there are still challenges to overcome in regard to oncolytic virotherapy, the combination regime analysed throughout this research has shown potentialities to induce immune response against the tumour, offering a glimmer of light to both melanoma and mesothelioma patients.

## Conclusions

Overall, our study reveal that combined treatment with AdV-D24-ICOSL-CD40L and anti-PD1 showed higher anti-tumour activity and a synergistic anti-cancer effect against both melanoma and mesothelioma animal models. Interestingly, as demonstrated in the presented research, anti-cancer effects positively correlated with CD8+cytotoxic T cell infiltrates, thus suggesting possible favourable therapeutic outcomes for cancer patients. Moreover, the development of novel oncolytic vectors armed with potent co-stimulatory molecules could induce durable anti-cancer immune responses and have important implications to advance further clinical therapeutic strategies for the treatment of solid malignancies such as melanoma and mesothelioma. The benefits of compounding various ICIs with oncolytic viruses-encoding many immunomodulatory molecules for patients with solid tumours may be a greater area of focus in the next years.

## Supplementary Material

Supplementary figures and table.

## Figures and Tables

**Table 1:** Treatment characteristics in immunocompetent mouse models.

#	Treatment group	Cell injection, both flanks	Dose	Schedule (days)
**Immunocompetent BALB/c AB12 xenograft mesothelioma mouse model**				
1	Mock (PBS)	1.5 × 10^7^ AB12 cells into both flanks of 10-week-old BALB/c male mice (6-10 tumours/group).	PBS i.t. and i.v.	1-6
2	AdV-D24-ICOSL-CD40L		1.75 × 10^10^ VP/tumour i.t.	
3	Anti-PD1		200 μg anti-PD1 i.v.	
4	AdV-D24-ICOSL-CD40L + Anti-PD1		1.75 × 10^10^ VP/tumour i.t. + 200 μg anti-PD1 i.v.	
**Immunocompetent C57BL/6 B16Vxenograft melanoma mouse model**				
1	Mock (PBS)	1 × 10^6^ B16V cells into both flanks of 10-week-old C57BL/6 male mice (6 tumours/group)	PBS i.t. and i.v.	1-6
2	AdV-D24-ICOSL-CD40L		1.75 × 10^10^ VP/tumour i.t.	
3	Anti-PD1		200 μg anti-PD1 i.v.	
4	AdV-D24-ICOSL-CD40L + Anti-PD1		1.75 × 10^10^ VP/tumour i.t. + 200 μg anti-PD1 i.v.	

**Table 2:** Assessment of therapeutic anti-cancer synergism in the AB12 and B16V models in immunocompetent mice (BALB/c and C57BL/6 respectively) with the fractional tumour volume (FTV) calculation method. The latest available tumour volume measurements have been taken for the purpose of the FTV calculation.

AB12 BALB/c mesothelioma immunocompetent mouse model					
Tumour growth	FTV		AdV-D24-ICOSL-CD40L + Anti-PD1		
	AdV-D24-ICOSL-CD40L	Anti-PD1	Exp^a^	Obs^b^	Ratio
			FTV	FTV	Exp/Obs
EoS	0.331	0.868	0.287	0.077	**3.718**
B16V C57BL/6 melanoma immunocompetent mouse model					
Tumour growth	FTV		AdV-D24-ICOSL-CD40L + Anti-PD1		
	AdV-D24-ICOSL-CD40L	Anti-PD1	Exp^a^	Obs^b^	Ratio
			FTV	FTV	Exp/Obs
EoS	0.284	0.922	0.261	0.212	**1.235**

^a^Exp, expected; ^b^Obs, observed; EoS, end of study/last measurements.
