# Supplementary material for: From Immunosuppression to Immunomodulation - Turning Cold Tumours into Hot
Source: J Cancer. 2022 Jul 4;13(9):2884–92. doi: 10.7150/jca.71992 (PMC9330456; doi:10.7150/jca.71992)
Supplement: Supplementary file 1 — Supplementary figures and table. [file jcav13p2884s1.pdf]

From Immunosuppression To Immunomodulation – Turning Cold Tumours Into Hot

Mariangela Garofalo[1,*], Katarzyna Wanda Pancer[2], Magdalena Wieczorek[2], Monika Staniszewska[3], Stefano Salmaso[1], Paolo Caliceti[1], Lukasz Kuryk[1,2*]

[1] Department of Pharmaceutical and Pharmacological Sciences, University of Padova, Via F. Marzolo 5, 35131 Padova, Italy
[2] Department of Virology, National Institute of Public Health NIH —National Institute of Research, Chocimska 24, 00-791 Warsaw, Poland
[3] Centre for Advanced Materials and Technologies, Warsaw University of Technology, Poleczki 19, Warsaw 02-822, Poland

* Correspondence: mariangela.garofalo@unipd.it (M.G.) & lkuryk@pzh.gov.pl (L.K.)

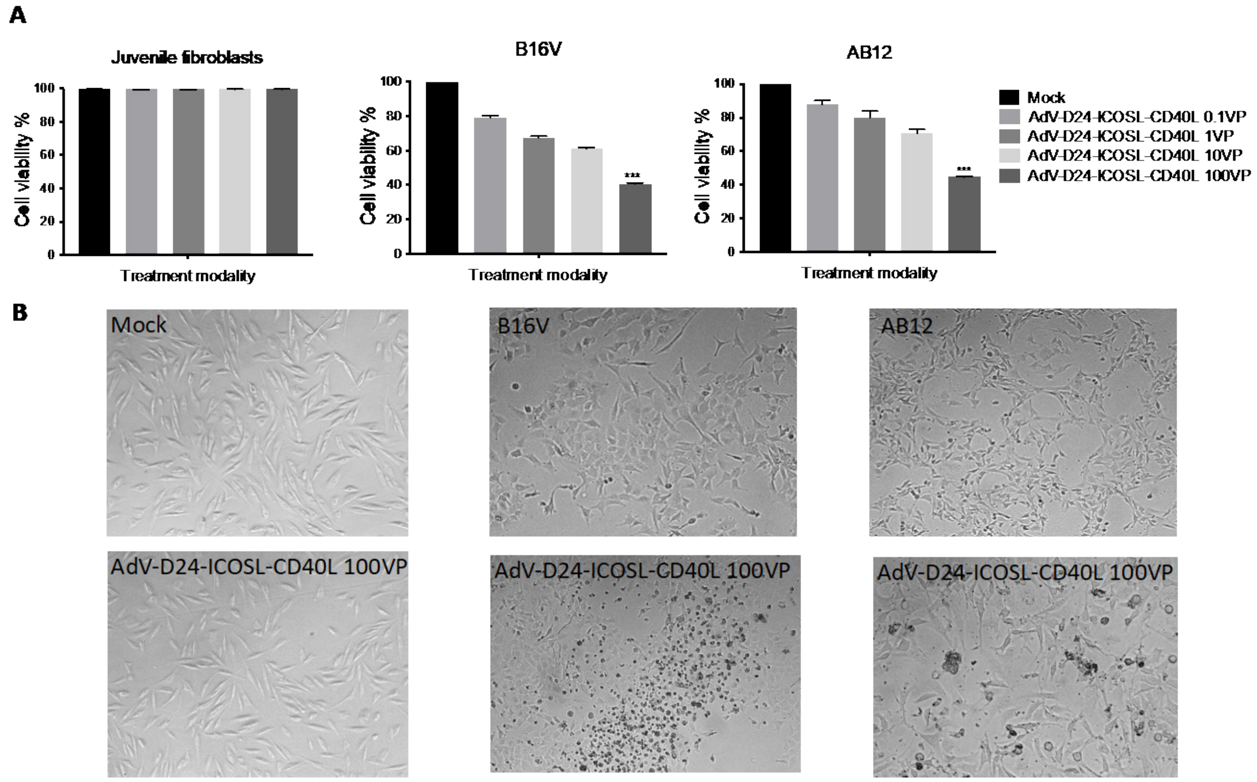

Figure S1. Evaluation of cell viability by MTS assay. **A** Cell viability was evaluated on fibroblasts, B16V murine melanoma and AB12 murine mesothelioma cells, 96 h post-infection with AdV-D24-ICOSL-CD40L at the concentration of 100, 10, 1, 0.1 VP/cell. Data are expressed as the percentage of viable cells according to MTS cell viability assay protocol (CellTiter 96® AQueous One Solution Cell Proliferation Assay, Promega). Statistical analysis was carried out with a Mann–Whitney test to compare two groups (*** = $p \leq 0.001$, vs mock). **B** Microscopic photographs visualizing the morphology and cytopathic effect (CPE) of the cells infected with AdV-D24-ICOSL-CD40L at the concentration of 100 VP/cell versus untreated control cells (Zeiss Axio Imager A2, Carl Zeiss Microscopy GmbH, Jena, Germany, magnification 10×).

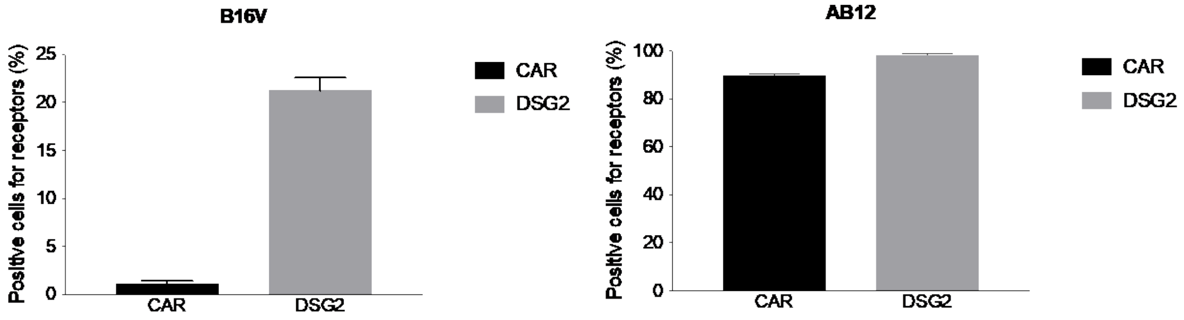

Figure S2. Expression of Coxsackie-Adenovirus Receptor (CAR) and Desmoglein-2 (DSG2) receptors in murine melanoma cell line B16V and murine mesothelioma cell line AB12, measured with flow cytometry (Beckman-Coulter Cytomics FC500). Data are expressed as percentage of cell positive for the marker.

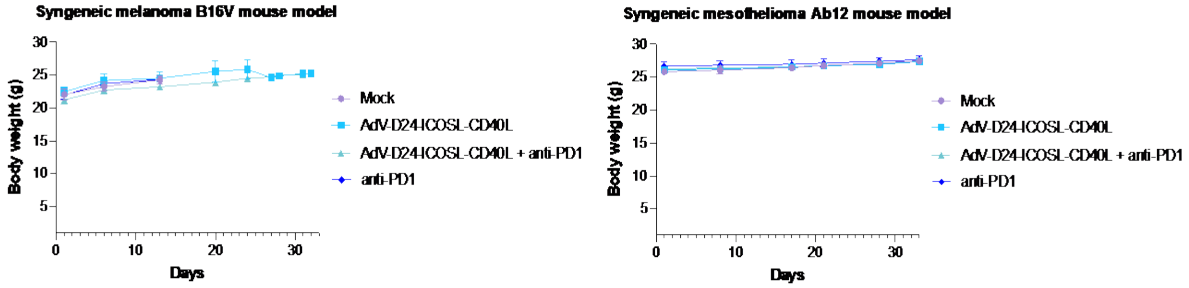

Figure S3. Body weight measurements throughout the study. BALB/c AB12 xenograft mesothelioma mouse model and C57BL/6 B16V xenograft melanoma mouse model. Mice were engrafted with $1x10^6$ cells/flank. The virus was administered intratumorally on days 1-6 i.t, anti-PD1 was given i.p. on days 1-6. The tumour volumes and weights, clinical health scores and body weight were monitored 2-3 times per week. Results are expressed as mean ± SEM.

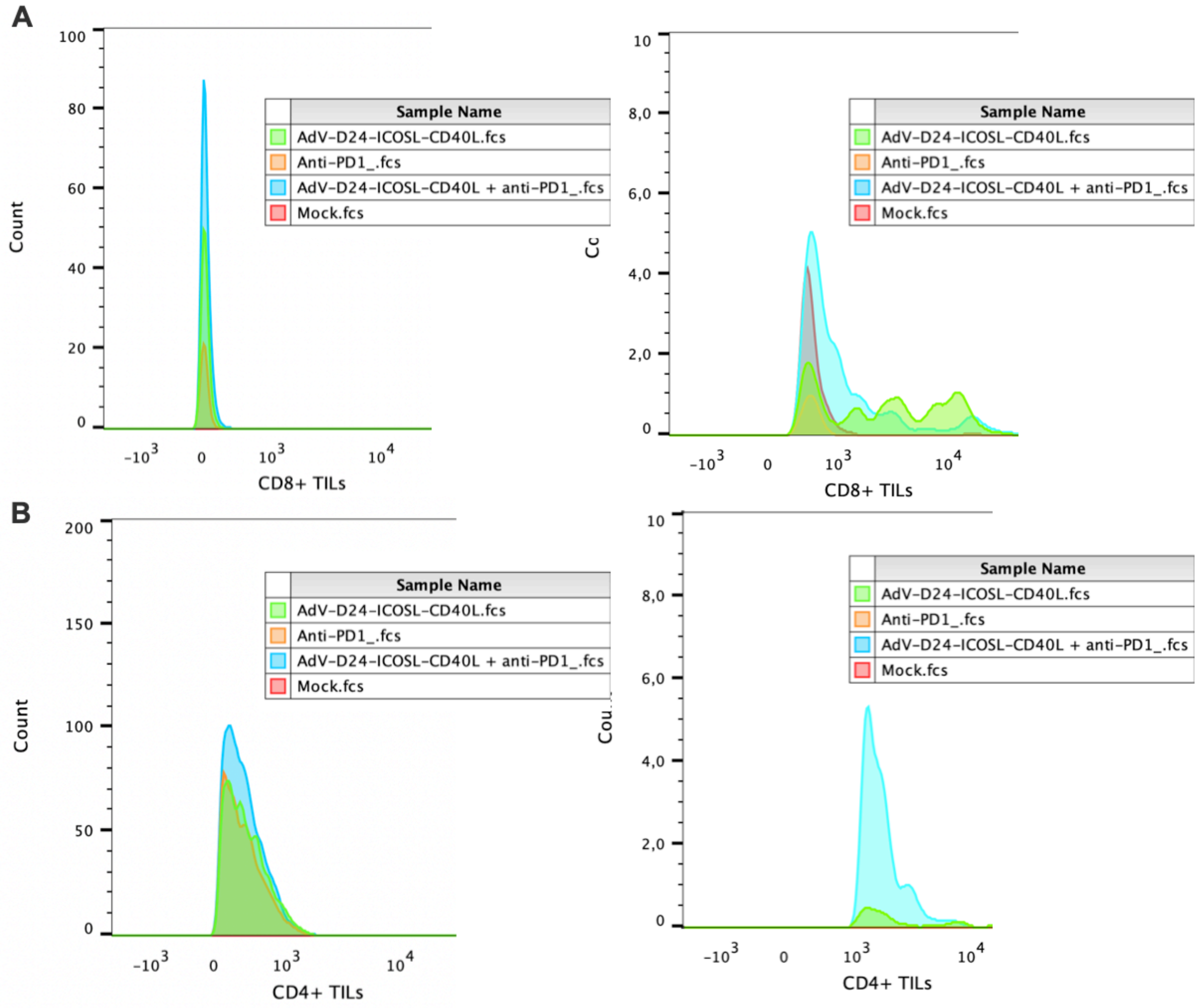

Figure S4. Flow cytometry histogram showing expression of CD8+ (**A**) and CD4+ (**B**) TILs. BALB/c AB12 xenograft mesothelioma mouse model (left panel). C57/BL6 B16V xenograft melanoma mouse model (right panel). Immune cells were isolated by following the protocol described earlier [32]. After dissociation, cells were washed and stained with antibodies 30 min at 4°C in the dark and then suspended in PBS. Samples were acquired using the BD FACSAria^{TM} III instrument. The populations were gated with forward and side scattering (FSC-A/SSC-A dot plot) in leukocytic regions (at least 10^4 cells/events were analyzed by flow cytometry). Samples were run in duplicates. Flow cytometry analysis was performed on FlowJo v10 software.

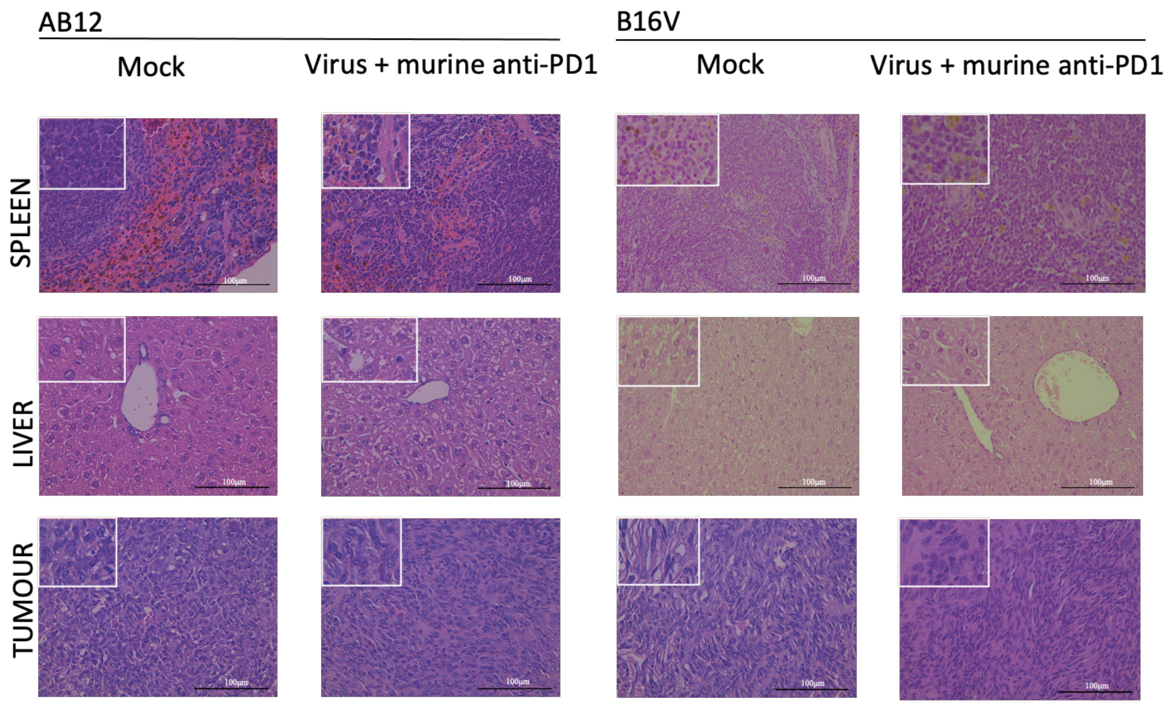

Figure S5. Histopathological examination of organs collected from mice in mock and treated with the virus plus anti-PD1 group: spleen, liver and tumour (BALB/c mice engrafted with AB12 mesothelioma cells and C57BL/6 mice engrafted with B16V melanoma cells). Histopathological analyses were performed using Haematoxylin and Eosin staining (Nuclei stained with DAPI (blue) (Scale bar: 100 µm, zoomed in area have been incorporated into each of the image – upper left corner). The histopathological evaluation was performed using the microscope Zeiss Axio Imager A2, Carl Zeiss Microscopy GmbH, Jena, Germany.

Table S1. Characteristics of clinical signs in animal health scoring.

Clinica sign	Abb.	Description	Score
**Coat**	C	Normal	0
		Lack of grooming, partial alopecia	1
		Massive alopecia, wounds, bleedings, inflammation	2
**Movement**	M	Normal	0
		Slow movements, paralysis of one member	2
		Difficulties to eat or drink, paralysis of more than one member	3
**Activity**	A	Normal	0
		Agitates, over-reactive hypo-reactive	1
		Prostrated	3
**Paleness**	P	Normal	0
		Slight	1
		Severe	2
**Body weight**	V	Normal	0
		Segmentation of the vertebral column evident, pelvic bones palpable	2
		Emaciated, skeletal structure prominent	3
